# The Association between Major Dietary Pattern and Low Muscle Mass in Korean Middle-Aged and Elderly Populations: Based on the Korea National Health and Nutrition Examination Survey

**DOI:** 10.3390/nu12113543

**Published:** 2020-11-19

**Authors:** Seong-Ah Kim, Jinwoo Ha, Byeonghwi Lim, Jun-Mo Kim, Sangah Shin

**Affiliations:** 1 Department of Food and Nutrition, Chung-Ang University, Gyeonggi-do 17546, Korea; sakim8864@gmail.com (S.-A.K.); vmffl1@naver.com (J.H.); 2 Department of Animal Science and Technology, Chung-Ang University, Gyeonggi-do 17546, Korea; hwi1208@cau.ac.kr (B.L.); junmokim@cau.ac.kr (J.-M.K.)

**Keywords:** dietary pattern, low muscle mass, skeletal muscle, sarcopenia

## Abstract

Reduced skeletal muscle mass in older populations is independently associated with functional impairment and disability, resulting in increased risk of mortality and various comorbidities. This study aimed to examine the association between major dietary pattern and low muscle mass among Korean middle-aged and elderly populations. A total of 8136 participants aged ≥50 years were included from a cross-sectional study based on the 2008–2011 Korea National Health and Nutrition Examination Survey. The following four distinct dietary patterns were derived using factor analysis: “Condiment, vegetables, and meats”; “wheat flour, bread, fruits, milk, and dairy products”; “white rice, fish, and seaweeds”; and “whole grain, bean products, and kimchi”. A higher “white rice, fish, and seaweeds” pattern score was associated with a lower prevalence of low muscle mass in both men and women, whereas a higher “condiment, vegetables, and meats” pattern score was associated with a higher prevalence of low muscle mass in men. A dietary pattern based on white rice, fish, and seaweeds can be helpful in protecting against loss of skeletal muscle mass in Korean middle-aged and elderly populations. Future research is paramount to confirm the causal association between dietary pattern and the risk of low muscle mass.

## 1. Introduction

Due to declining fertility rates and remarkable increases in life expectancy, the number of people aged 65 years or older is projected to grow from an estimated 524 million in 2010 to nearly 1.5 billion in 2050, globally [1]. Population aging is one of the most important global public health problems in modern society. Aging naturally involves various physiological changes, such as a decrease in skeletal muscle mass and increase in fat mass [2]. Reduced skeletal muscle mass in older populations is independently associated with functional impairment and disability, sequentially resulting in increased risk of mortality and various comorbidities [3,4,5,6].

Sarcopenia is a complex geriatric syndrome that is associated with various adverse health outcomes, such as physical disability, poor quality of life, and death [7,8]. Previously, loss of muscle mass associated with aging was commonly used to define sarcopenia [9]; however, more recent definitions have recommended using the presence of both low muscle mass and low muscle function (strength or performance) for the diagnosis of sarcopenia [10,11]. In brief, sarcopenia is characterized by progressive loss of skeletal muscle mass and strength; hence, low muscle mass is a sufficient condition for sarcopenia.

Sarcopenia and loss of muscle mass can arise from multifactorial pathogenesis, including neuromuscular degeneration, changes in muscle protein turnover, changes in hormone levels and sensitivity, chronic inflammation, oxidative stress, and behavior/lifestyle factors [8]. Among these risk factors, lifestyle factors, such as physical activity and diet, are the most reversible and modifiable [8]. Exercise (both resistance and aerobic), in combination with nutritional strategy, has been recommended as a key component in the prevention and management of sarcopenia [12]. In particular, the main nutritional strategies proposed for the treatment of sarcopenia or low muscle mass include the increased intakes of protein; vitamin D supplementation; and antioxidants, such as selenium, vitamin A, vitamin C, vitamin E, and β-carotene [8].

Because people consume holistic meals, rather than single-nutrient or single-food diets, on a daily basis, the dietary pattern approach is often recommended to examine the association between diet and diet-related disease. The dietary pattern approach has been widely used to examine intricate interactions of nutrients and foods with various diseases as well as their synergistic effects on the same [13]. Dietary patterns conceptually indicate a broader picture of the overall diet; therefore, examining dietary patterns may be more predictive of disease risk than individual nutrients or foods [13].

Population aging in Korea is expected to accelerate sharply in the next three decades, faster than in other Organization for Economic Co-operation and Development (OECD) member countries [14]. In addition, Korean people who traditionally consume rice-based high-carbohydrate, low-protein, and low-fat diets may be more prone to low muscle mass. In this respect, establishing a nutritional strategy that prevents and manages age-related health problems of the elderly population is one of the most practical and effective plans to alleviate the social burden of the elderly society in the future. In particular, understanding the dietary pattern of people with higher or lower risk of low muscle mass can provide evidence to develop practical guidelines for preventing and managing low muscle mass. Therefore, in the present study, we aimed to examine the association between major dietary pattern and low muscle mass among Korean middle-aged and elderly populations.

## 2. Materials and Methods

### 2.1. Study Population

**Figure 1:**
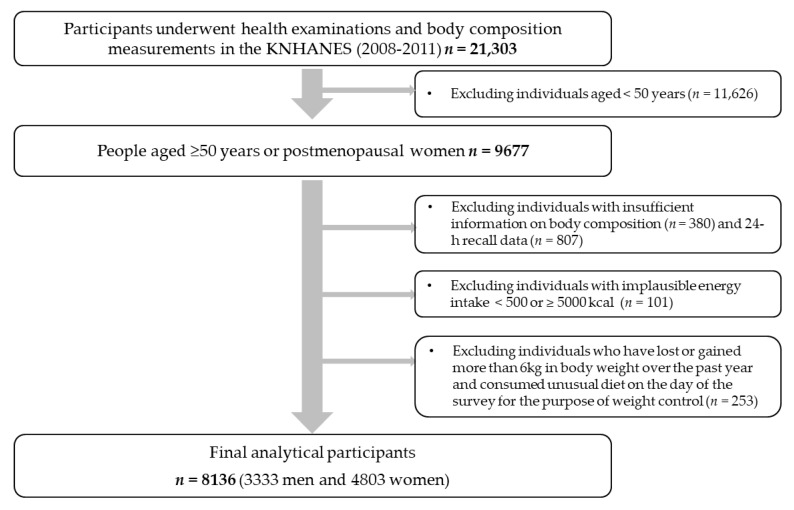
Flow diagram of the selection of study participants: the KNHANES (2008–2011).

The data used in the current study were based on the 2008–2011 Korea National Health and Nutrition Examination Survey (KNHANES). The KNHANES is a cross-sectional, nationally representative survey in South Korea annually conducted by the Korea Centers for Disease Control and Prevention (KCDC). It consists of a health examination survey, health behavioral survey, and nutritional survey, and it occasionally involves body composition measurements. A flow diagram of the selection of study participants is shown in Figure 1. To date, body composition measurements were conducted between 2008 and 2011. Among participants who underwent health examinations and body composition measurements (*n* = 21,303), those aged ≥50 years or postmenopausal women were selected (*n* = 9677). Among them, we excluded those who did not have sufficient information on body composition (*n* = 380) and 24-h recall data (*n* = 807). We further excluded those with implausible energy intakes (<500 or >5000 kcal/day; *n* = 101) and those who had lost or gained more than 6kg in body weight over the past year and consumed an unusual diet on the day of the survey for the purpose of weight control (*n* = 253). Finally, a total of 8136 participants (3333 men and 4803 women) were included in this study. The present study was conducted according to the guidelines laid down in the Declaration of Helsinki. All procedures involved in the KNHANES were approved by the KCDC Institutional Review Board (IRB No: 2008-04EXP-01-C, 2009-01CON-03-2C, 2010-02CON-21-C, and 2011-02CON-06-C), and all participants provided written informed consent.

### 2.2. Dietary Assessment and Dietary Pattern Analysis

**Figure 2:**
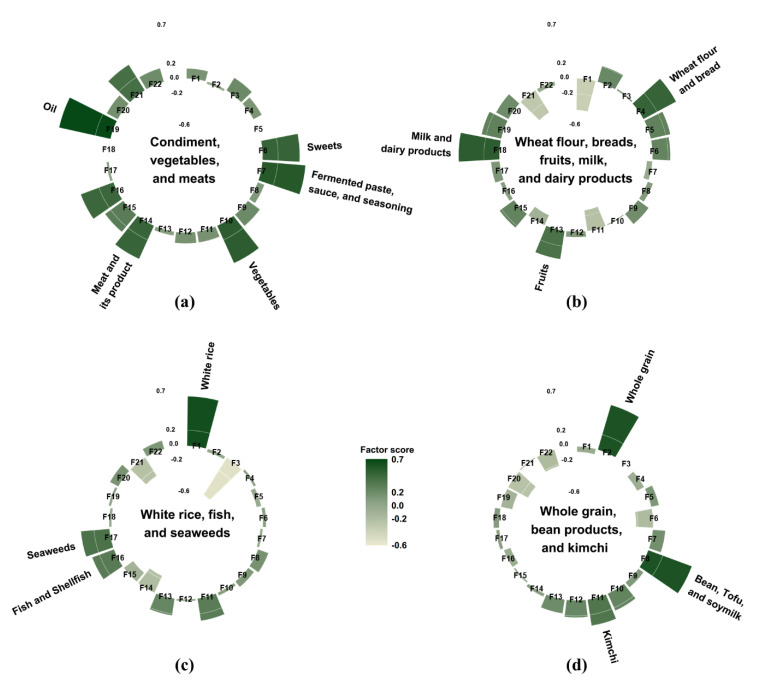
Factor loading matrix of food groups for the four dietary patterns identified from the 24-h dietary recall. (**a**) the “condiment, vegetables, and meats” pattern; (**b**) the “wheat flour, breads, fruits, milk, and dairy products” pattern; (**c**) the “white rice, fish, and seaweeds” pattern; and (**d**) the “whole grain, bean products, and kimchi” pattern. The top three food groups with the highest factor loading value were used to name each dietary pattern. As an exception, oils, fermented paste, sauce, and seasoning, and sweets were grouped into condiments and used for naming the “condiment, vegetables, and meats” pattern. F1: white rice, F2: whole grain, F3: noodles and dumpling, F4: wheat flour and bread, F5: potatoes and corn, F6: sweets, F7: fermented paste, sauce, and seasoning, F8: bean, tofu, and soymilk, F9: nuts and seeds, F10: vegetables, F11: kimchi, F12: mushroom, F13: fruits, F14: meat and its product, F15: eggs, F16: fish and shellfish, F17: seaweeds, F18: milk and dairy products, F19: oil, F20: beverage, F21: alcohol, F22: coffee and tea.

Dietary intake was assessed using a one-day 24-h dietary recall method. Nutrient-intake calculations were based on the KNHANES nutrient database. In total, 657 food items were reported in this study, and they were categorized into 22 food groups based on the Korean Nutrition Database and previous studies on dietary patterns in Korea [15,16] (Figure 2). Since white rice is the staple food of Koreans, it was isolated from the grain group. Similarly, since kimchi is typically one of the most frequently consumed foods in Korea, it was also separated from other vegetables. The average energy intake of each of the 22 food groups was calculated. We subsequently conducted a factor analysis to identify the major dietary patterns based on the total daily energy intake from the 22 food groups. The factors were rotated by an orthogonal transformation to achieve a simpler structure with greater interpretability. To determine the number of factors, we considered eigenvalues, scree plot, and the interpretability of the derived patterns. The derived patterns were named from foods loaded most positively on each pattern. Participants had their own factor score for each identified pattern, and they were categorized by sex into tertiles for every pattern.

### 2.3. Body Composition Measurement

Body weight (kg) and height (cm) were measured according to standard protocol, with the participant in light clothing and without shoes. Body mass index (BMI) was calculated as follows: weight divided by the height squared (kg/m^2^).

Body composition was measured using dual-energy X-ray absorptiometry (DEXA, Discovery QDR 4500; Hologic, Inc., Waltham, MA, USA) at the health examination center on the same day when the participants took anthropometric measurement and blood test after 8 h of fasting. Appendicular skeletal muscle mass (ASMM, kg) was calculated as the sum of muscle mass in the arms and legs, assuming that all non-fat and non-bone tissue is skeletal muscle, in accordance with previous studies [17,18,19]. According to the definition proposed in a previous study [20], we used the weight-adjusted ASMM (ASMM/weight × 100 kg/m^2^). To establish the cut-off value for low muscle mass, the gender-specific mean (33.92 in men, 26.35 in women) and standard deviation (SD) (2.72 in men, 2.30 in women) of the weight-adjusted ASMM of the young-adult reference group (healthy men and women aged 20–39 years) were used. Individuals whose weight-adjusted ASMM was higher than 1SD below the gender-specific mean for the young reference group were considered normal according to each definition. Class I low muscle mass was indicated by definition in participants whose weight-adjusted ASMM was between the gender-specific mean for the young reference group, 1 SD, and the mean for young adults, 2 SD. Class II low muscle mass was indicated by definition in participants whose weight-adjusted ASMM was below the mean for young adults, 2 SD [3].

### 2.4. Assessment of Other Variables

Demographic characteristics, including age and household income, were acquired from a self-report questionnaire. Household income was categorized into quartiles. Lifestyle factors included smoking (never smoked, past smoker, current smoker), alcohol drinking (current alcohol drinker, non-alcohol drinker), and physical activity.

Physical activity was estimated using the Korean version of the International Physical Activity Questionnaire (IPAQ) [21]. The duration (in minutes) of physical activity was then converted into metabolic equivalents (MET) [22], and our participants were subsequently categorized into the following 3 groups on the basis of the IPAQ guidelines: “High”, “moderate”, or “low” [23]. The “high” group included (i) those who performed vigorous-intensity activity at least three days a week, achieving a minimum total physical activity of 1500 MET-min/week or (ii) those who engaged in seven or more days of any combination of walking, moderate-intensity, or vigorous-intensity activities, achieving a minimum total physical activity of 3000 MET-min/week. The “moderate” group comprised individuals who performed (i) three or more days of vigorous-intensity activity of at least 20 min/day, (ii) five or more days of moderate-intensity activity and/or walking of at least 30 min/day, or (iii) five or more days of any combination of walking, moderate-intensity, or vigorous-intensity activities, achieving a minimum total physical activity of 600 MET-min/week. Individuals who did not meet the criteria for the aforementioned categories were placed in the “low” group.

The serum 25-hydroxyvitamin D (25[OH]D) level was measured by radioimmunoassay (DiaSorin Inc., Stillwater, MN, USA) using a gamma counter (1470 Wizard; PerkinElmer, Turku, Finland).

### 2.5. Statistical Analysis

The participants were divided into tertile groups according to each of the four dietary pattern scores. Differences in the general characteristics of participants across the tertile groups of each dietary pattern score were determined using the chi-square test for categorical variables and the generalized linear model for continuous variables. Multivariable-adjusted logistic regression analysis was conducted to compare the odds ratios (ORs) and 95% confidence intervals (CIs) for low muscle mass among tertiles of each dietary pattern score. Logistic regression models were adjusted for age (continuous), BMI (continuous), energy intake (continuous), smoking status (never, past, and current), alcohol consumption (non-current, current), physical activity (low, moderate, and high), household income (low, middle-low, middle-high, and high), and serum vitamin D (continuous). In general, because the correlation between educational and household income levels was high, only household income level was selected as an adjustment variable. All statistical analyses were performed using SAS version 9.4 (SAS Institute Inc., Cary, NC, USA). Statistical significance was defined as *p* < 0.05.

## 3. Results

Four major dietary patterns were identified from the factor analysis (Figure 2), and they were named after foods or food groups with high factor loading values. These four dietary patterns accounted for 27.8% of the total variance: 10.0%, 6.5%, 6.0%, and 5.3%, respectively. The “condiment, vegetables, and meats” pattern was characterized by a high consumption of condiments, including oil, sweets, fermented paste, sauce, and seasoning as well as vegetables, meat and its products, fish and shellfish, eggs, and alcohol. The “wheat flour, breads, fruits, milk, and dairy products” pattern had positive factor loadings for milk and dairy products, wheat flour and breads, fruits, potatoes and corn, oil, sweets, eggs, and whole grains; however, it had negative loadings for white rice, kimchi, and alcohol. The “white rice, fish, and seaweeds” pattern featured a high consumption of white rice, fish, seaweeds, kimchi, and fruits as well as a low consumption of noodles and dumplings, meat and its products, and alcohol. The “whole grain, bean products, and kimchi” pattern showed high positive factor loadings for whole grain, bean products, kimchi, vegetables, and mushroom as well as negative factor loadings for beverages, coffee and tea, and sweets.

The general characteristics of study participants according to the tertiles of each dietary pattern score by sex are shown in Table 1. People with higher pattern scores for the “condiment, vegetables, and meats” pattern tended to be younger, have higher body fat mass and % body fat, have higher household income levels, and be alcohol drinkers compared to those with lower pattern scores (all *p* < 0.01). A higher “wheat flour, breads, fruits, milk, and dairy products” pattern score was associated with participants’ higher body fat mass as well as higher household income levels (all *p* < 0.05). People with higher “white rice, fish, and seaweeds” pattern scores were more likely to have lower body fat mass and % body fat compared to those with lower pattern scores (all *p* < 0.01). People with higher pattern scores for the “whole grain, bean products, and kimchi” pattern tended to have lower % body fat, be currently non-smokers, earners of higher household income levels, and more physically active compared to those with lower pattern scores (all *p* < 0.01).

Table 2 displays nutrient intakes of study participants according to the tertiles of each dietary pattern score by sex. The “condiment, vegetables, and meats” and “whole grain, bean products, and kimchi” pattern scores were positively associated with energy intake and other nutrient intakes, including percentage of energy from protein and fat, fiber, calcium, phosphorus, iron, sodium, potassium, vitamin A, carotenoid, thiamin, riboflavin, niacin, and vitamin C, except percentage of energy from carbohydrates (all *p* < 0.001). The “wheat flour, breads, fruits, milk, and dairy products” pattern score was positively associated with the percentage of energy intake from protein and fat (all *p* < 0.001), whereas the “white rice, fish, and seaweeds” pattern score was positively associated with percentage of energy from carbohydrates, fiber, calcium, iron, carotenoid, and vitamin C intake (all *p* < 0.001).

Table 3 shows the ORs and 95% CIs for risk of low muscle mass according to the tertiles of each dietary pattern score by sex. In men, a higher “condiment, vegetables, and meats” pattern score was associated with a higher OR for class II low muscle mass (OR 1.51, 95% CI 1.05–2.18, *p* for trend = 0.030). On the other hand, a higher “white rice, fish, and seaweeds” pattern score was associated with a lower OR for class I low muscle mass in both men and women (men: OR 0.72, 95% CI 0.59–0.88, *p* for trend = 0.001; women: OR 0.76, 95% CI 0.63–0.91, *p* for trend = 0.003). No significant association was observed between each of the “wheat flour, breads, fruits, milk, and dairy products” and “whole grain, bean products, and kimchi” patterns and low muscle mass.

## 4. Discussion

This cross-sectional study, conducted on a representative sample in Korea, showed that a higher “white rice, fish, and seaweeds” pattern score was associated with a lower prevalence of low muscle mass in both men and women, whereas a higher “condiment, vegetables, and meats” pattern score was associated with a higher prevalence of low muscle mass in men.

In the present study, the following four major dietary patterns were derived from Korean middle-aged and elderly populations: the “condiment, vegetables, and meats” pattern; “wheat flour, breads, fruits, milk, and dairy products” pattern; “white rice, fish, and seaweeds” pattern; and “whole grain, bean products, and kimchi” pattern. These dietary patterns were distinguished from those of previous Korean studies that examined the association between dietary pattern and muscle mass or muscle strength [24,25]. In a previous cross-sectional study that investigated the association between dietary patterns and ASMM in the Korean older population, two dietary patterns were identified: “Healthy” and “Western” [24]. The “healthy” dietary pattern, which was characterized by a high intake of vegetables, fish, fruits, seaweed, legumes, mushrooms, whole grains, potatoes, eggs, dairy products, and red meat, was associated with a higher ASMM in men aged ≥60 years [24]. Moreover, another cross-sectional study that investigated the association between dietary patterns and handgrip strength identified two dietary patterns: “Prudent” and “Western” [25]. The study revealed a significantly positive association between handgrip strength and the “prudent” dietary pattern, characterized by higher intakes of vegetables, potatoes, fish, mushroom, fruits, nuts, legumes, and mixed grains [25]. The differences in derived dietary patterns and their associations with muscle mass or muscle strength between previous studies and the present one may be primarily due to the difference in dietary assessment methods. Previous studies used food frequency questionnaires (FFQs) to assess the participants’ diets, whereas the present study used the 24-h dietary recall method. The FFQ consists of a predefined list of foods, whereas 24-h dietary recall is open-ended [26]. Open-ended questions, such as those in 24-h dietary recall, may be more appropriate for collecting a variety of detailed dietary information from participants than closed-ended ones, such as those in the FFQ [26], since the dietary pattern approach focuses on the combined and synergetic effects of various foods.

This study found that the “white rice, fish, and seaweeds” pattern was associated with a lower prevalence of low muscle mass in both men and women. Additionally, the mean appendicular skeletal muscle mass of the highest “white rice, fish, and seaweeds” pattern score group (men: 20.7 kg, women: 14.0 kg) is higher than that of the lowest pattern score group (men: 20.5 kg, women: 13.9 kg), though it was not statistically significant. The underlying mechanism can be explained by the role of various nutrients predominant in fish and seaweeds, such as protein, *n*-3 polyunsaturated fatty acid (PUFA), vitamin D, magnesium, and carnitine, which can be involved positively in muscle metabolism [27]. First, protein intake is crucial for maintaining skeletal muscle because skeletal muscle is the main reservoir of amino acids, containing 50–75% of all proteins in the human body [28]. Amino acids, especially leucine and insulin-like growth factor 1, are anabolic stimuli for muscle metabolism [29]. Regarding the protein source, fish protein can increase skeletal muscle weight in rats [30]; however, very few studies have focused on the effects of fish proteins on muscle mass in humans. Second, *n*-3 PUFA, predominant in fish, can mediate an increase in muscle protein synthesis. It is incorporated into the cellular membranes of various body tissues, including skeletal muscle, and may enhance membrane fluidity, thus improving the uptake of amino acids and, consequently, making the cells more responsive to muscle protein synthesis [31,32]. Finally, combining several anabolic nutrients, such as amino acids, *n*-3 PUFA, vitamin D, and magnesium, could increase protein synthesis and finally promote muscle mass gain [33,34].

In the present study, the “condiment, vegetables, and meats” pattern was associated with a higher prevalence of class II low muscle mass in men. Men with a higher “condiment, vegetables, and meats” pattern score (2703.1 kcal/day) consumed 1.8 times more calories per day than those with a lower one (1522.7 kcal/day). Moreover, men in the highest “condiment, vegetables, and meats” pattern score group had higher BMI, body fat mass, and % body fat, and higher prevalence of obesity compared to the lowest group (Table 1). This excessive calorie intake and higher BMI, body fat mass, and % body fat, and higher prevalence of obesity suggests the possibility of obesity in people who adhere to the “condiment, vegetables, and meats” pattern. Thus, low muscle mass is possibly associated with sarcopenic obesity, which involves the coexistence of age-related physical changes, such as a decrease in skeletal muscle mass and function (sarcopenia) as well as an increase in fat mass (obesity) [34]. In addition to sarcopenia and obesity having similar pathological pathways, obesity could be a risk factor for the development of sarcopenia [35]. The increase in intramuscular fat involved in obesity can affect the signaling pathways that are engaged in muscle protein synthesis and thereby increase the risk for sarcopenia [34,36].

In this study, the “condiment, vegetables, and meats” pattern was associated with an increased prevalence of class II low muscle mass in men, not in women. This sex difference can be explained by the unhealthy lifestyle of men compared to women. The highest “condiment, vegetables, and meats” pattern score group in men showed a much higher percentage of current smokers (men: 35.5%, women: 4.1%) and current drinkers (men: 74.2%, women: 31.1%) than that of women (Table 1). The results of a meta-analysis demonstrate that cigarette smoking is an isolated factor, which may contribute to the development of sarcopenia [37]. Alcohol consumption has not been reported as a direct cause of sarcopenia; however, previous evidence suggests that chronic alcohol consumption may promote loss of muscle mass in older people [38]. Although these lifestyle factors were statistically adjusted in the present analysis, these unhealthy lifestyles might exert combined negative effects on low muscle mass in men.

Findings from the present study showed that people adhering to the “white rice, fish, and seaweeds” pattern were less likely to have low muscle mass, whereas men who adhered to the “condiment, vegetables, and meats” pattern were more likely to have low muscle mass. To prevent and manage age-related loss of skeletal muscle mass, it may be recommended to adhere to a dietary pattern based on white rice, fish, and seaweed and avoid one with excessive calories, such as that consisting of mainly oil, sugar, and various sauces or seasoning. To design nutritional strategies that prevent low muscle mass, dietary patterns that ensure optimal nutrient intake should be developed to not only increase skeletal muscle mass or prevent muscle mass loss but also decrease excess fat mass, thereby preventing sarcopenic obesity. Additionally, to maximize the benefits of dietary pattern on muscle mass, adequate physical activity should be taken into consideration [38].

To the best of our knowledge, this study is the first to use 24-h dietary recall in investigating the association between major dietary pattern and the prevalence of low muscle mass among Korean middle-aged and elderly populations. Compared to previous studies that used the FFQ to derive dietary patterns among the Korean older population, the present one could derive more detailed and distinctive dietary patterns of Korean middle-aged and elderly populations.

However, several limitations should be considered when interpreting the results. First, due to the nature of the cross-sectional design, causal association between dietary pattern and the risk of low muscle mass cannot be confirmed from the present study’s results. Second, using the one-day 24-h dietary recall method may not reflect the participant’s usual intake. Finally, the factor analysis approach entails arbitrary yet important decisions in some processes, including the classification of food items into food groups, the number of factors to derive, the method of factor rotation, and even the labelling of the derived factors [39]. Therefore, the dietary pattern approach can be somewhat subjective, and the results may be difficult to replicate in other populations. Future research will be needed to confirm the causal association between dietary pattern and the risk of low muscle mass or muscle function in a large-scaled prospective cohort or randomized clinical trials.

## 5. Conclusions

The large-scaled cross-sectional study in Korea demonstrated that a dietary pattern based on white rice, fish, and seaweeds is associated with a lower prevalence of low muscle mass in middle-aged and elderly populations. Further, the aforementioned dietary pattern can be helpful in protecting against loss of skeletal muscle mass in the same populations.

## Figures and Tables

**Table 1:** General characteristics of study participants according to the tertiles of each dietary pattern score by sex.

	Tertile of Each Pattern Score							
	Men				Women			
	T1	T2	T3	*p* Value ^1^	T1	T2	T3	*p* Value ^1^
Condiment, vegetables, and meats								
Age (year)	67.3 ± 8.6	63.9 ± 8.2	60.8 ± 7.8	<0.001	66.1 ± 9.2	62.9 ± 9.3	60.1 ± 8.6	<0.001
BMI (kg/m^2^)	23.2 ± 3.0	23.6 ± 2.9	24.2 ± 2.8	<0.001	24.1 ± 3.3	24.3 ± 3.3	24.3 ± 3.1	0.090
Obesity prevalence, *n* (%)	300 (27.0)	352 (31.7)	404 (36.4)	<0.001	582 (36.4)	609 (38.0)	608 (38.0)	0.535
Lean body mass (kg) ^2^	50.4 ± 0.3	50.6 ± 0.3	50.4 ± 0.3	0.367	36.9 ± 0.2	37.1 ± 0.2	36.8 ± 0.2	0.037
Appendicular skeletal muscle mass (kg) ^2^	20.5 ± 0.2	20.6 ± 0.2	20.5 ± 0.2	0.424	14.0 ± 1.0	14.1 ± 1.0	13.9 ± 1.0	0.018
Body fat mass (kg) ^2^	14.7 ± 0.2	14.9 ± 0.2	15.3 ± 0.2	<0.001	19.4 ± 0.2	19.5 ± 0.2	19.8 ± 0.2	0.004
% Body fat (%) ^2^	22.2 ± 0.3	22.3 ± 0.3	22.8 ± 0.3	0.005	33.9 ± 0.2	33.9 ± 0.2	34.4 ± 0.3	0.001
Serum vitamin D (ng/mL)	20.8 ± 7.3	21.5 ± 7.5	21.5 ± 7.0	0.033	18.7 ± 7.0	18.6 ± 6.8	18.7 ± 6.8	0.682
Household income ^3^, *n* (%)								
Low	476 (42.8)	317 (28.5)	205 (18.5)	<0.001	721 (45.0)	566 (35.4)	406 (25.4)	<0.001
Middle-low	297 (26.7)	310 (27.9)	266 (23.9)		377 (23.6)	438 (27.4)	391 (24.4)	
Middle-high	179 (16.1)	237 (21.3)	290 (26.1)		255 (15.9)	302 (18.9)	369 (23.1)	
High	145 (13.1)	240 (21.6)	339 (30.5)		223 (13.9)	268 (16.7)	414 (25.9)	
Current smoking status ^3^								
Non-smoker	213 (19.2)	184 (16.6)	165 (14.9)	0.003	1426 (89.1)	1473 (92.0)	1470 (91.8)	0.057
Past smoker	575 (51.8)	574 (51.7)	547 (49.2)		80 (5.0)	58 (3.6)	56 (3.5)	
Current smoker	312 (28.1)	348 (31.3)	394 (35.5)		78 (4.9)	57 (3.6)	66 (4.1)	
Alcohol consumption ^3^								
Non-drinker	486 (43.7)	342 (30.8)	279 (25.1)	<0.001	1274 (79.6)	1189 (74.3)	1088 (68.0)	<0.001
Current drinker	611 (55.0)	763 (68.7)	824 (74.2)		305 (19.1)	396 (24.7)	498 (31.1)	
Physical activity ^3^								
Low	451 (40.6)	436 (39.2)	456 (41.0)	0.005	728 (45.5)	775 (48.4)	765 (47.8)	0.104
Moderate	359 (32.3)	369 (33.2)	319 (28.7)		552 (34.5)	516 (32.2)	495 (30.9)	
High	285 (25.7)	302 (27.2)	331 (29.8)		298 (18.6)	296 (18.5)	328 (20.5)	
Wheat flour, breads, fruits, milk, and dairy products								
Age (year)	64.0 ± 8.4	65.0 ± 8.7	63.1 ± 8.5	<0.001	65.5 ± 9.2	63.4 ± 9.2	60.2 ± 8.9	<0.001
BMI (kg/m^2^)	23.5 ± 3.0	23.5 ± 2.9	24.0 ± 2.9	<0.001	24.1 ± 3.4	24.3 ± 3.2	24.2 ± 3.1	0.121
Obesity prevalence, *n* (%)	336 (30.2)	326 (29.3)	394 (35.5)	0.004	589 (36.8)	632 (39.5)	578 (36.1)	0.114
Lean body mass (kg)	50.3 ± 0.3	50.5 ± 0.3	50.4 ± 0.3	0.688	36.7 ± 0.2	36.9 ± 0.2	37.2 ± 0.2	0.002
Appendicular skeletal muscle mass (kg)	20.5 ± 0.2	20.6 ± 0.2	20.5 ± 0.2	0.613	13.9 ± 1.0	14.0 ± 1.0	14.1 ± 1.0	0.024
Body fat mass (kg)	14.8 ± 0.2	15.0 ± 0.2	15.1 ± 0.2	0.018	19.2 ± 0.2	19.6 ± 0.2	19.8 ± 0.2	<0.001
% Body fat (%)	22.2 ± 0.3	22.5 ± 0.3	22.6 ± 0.3	0.099	33.7 ± 0.2	34.2 ± 0.2	34.2 ± 0.2	<0.001
Serum vitamin D (ng/mL)	22.2 ± 7.7	21.0 ± 7.2	20.6 ± 6.8	<0.001	19.0 ± 7.0	18.4 ± 6.9	18.6 ± 6.7	0.024
Household income, *n* (%)								
Low	386 (34.7)	352 (31.7)	260 (23.4)	<0.001	715 (44.7)	599 (37.4)	379 (23.7)	<0.001
Middle-low	310 (27.9)	296 (26.6)	267 (24.0)		407 (25.4)	413 (25.8)	386 (24.1)	
Middle-high	196 (17.6)	242 (21.8)	268 (24.1)		254 (15.9)	306 (19.1)	366 (22.9)	
High	212 (19.1)	207 (18.6)	305 (27.5)		196 (12.2)	260 (16.2)	449 (28.0)	
Current smoking status								
Non-smoker	150 (13.5)	195 (17.6)	217 (19.5)	<0.001	1445 (90.3)	1456 (90.9)	1468 (91.7)	0.546
Past smoker	534 (48.1)	578 (52.0)	584 (52.6)		66 (4.1)	64 (4.0)	64 (4.0)	
Current smoker	422 (38.0)	328 (29.5)	304 (27.4)		79 (4.9)	67 (4.2)	55 (3.4)	
Alcohol consumption								
Non-drinker	260 (23.4)	405 (36.5)	442 (39.8)	<0.001	1185 (74.0)	1198 (74.8)	1168 (73.0)	0.498
Current drinker	845 (76.1)	694 (62.5)	659 (59.3)		403 (25.2)	384 (24.0)	412 (25.7)	
Physical activity								
Low	445 (40.1)	454 (40.9)	444 (40.0)	0.001	769 (48.0)	779 (48.7)	720 (45.0)	0.202
Moderate	314 (28.3)	374 (33.7)	359 (32.3)		500 (31.2)	519 (32.4)	544 (34.0)	
High	348 (31.3)	269 (24.2)	301 (27.1)		318 (19.9)	283 (17.7)	321 (20.1)	
White rice, fish, and seaweeds								
Age (year)	63.3 ± 8.7	65.2 ± 8.7	63.5 ± 8.3	<0.001	62.6 ± 9.6	63.8 ± 9.4	62.8 ± 9.0	<0.001
BMI (kg/m^2^)	23.7 ± 2.9	23.5 ± 2.9	23.8 ± 2.9	0.209	24.1 ± 3.2	24.2 ± 3.2	24.3 ± 3.2	0.579
Obesity prevalence, *n* (%)	354 (31.9)	333 (30.0)	369 (33.2)	0.257	600 (37.5)	604 (37.7)	595 (37.2)	0.947
Lean body mass (kg)	50.3 ± 0.3	50.5 ± 0.3	50.6 ± 0.3	0.126	36.9 ± 0.2	36.9 ± 0.2	37.0 ± 0.2	0.730
Appendicular skeletal muscle mass (kg)	20.5 ± 0.2	20.6 ± 0.2	20.7 ± 0.2	0.059	13.9 ± 1.0	14.0 ± 1.0	14.0 ± 1.0	0.330
Body fat mass (kg)	15.2 ± 0.2	15.0 ± 0.2	14.7 ± 0.2	<0.001	19.7 ± 0.2	19.5 ± 0.2	19.4 ± 0.2	0.005
% Body fat (%)	22.7 ± 0.3	22.5 ± 0.3	22.0 ± 0.3	<0.001	34.3 ± 0.2	34.1 ± 0.2	33.8 ± 0.2	0.009
Serum vitamin D (ng/mL)	21.2 ± 7.2	21.3 ± 7.3	21.5 ± 7.4	0.653	18.4 ± 6.7	18.7 ± 7.0	18.8 ± 6.9	0.212
Household income, *n* (%)								
Low	317 (28.5)	360 (32.4)	321 (28.9)	0.104	540 (33.7)	599 (37.4)	554 (34.6)	0.010
Middle-low	269 (24.2)	296 (26.6)	308 (27.7)		398 (24.9)	409 (25.6)	399 (24.9)	
Middle-high	247 (22.2)	227 (20.4)	232 (20.9)		320 (20.0)	273 (17.1)	333 (20.8)	
High	266 (23.9)	216 (19.4)	242 (21.8)		325 (20.3)	285 (17.8)	295 (18.4)	
Current smoking status								
Non-smoker	168 (15.1)	188 (16.9)	206 (18.5)	<0.001	1427 (89.1)	1463 (91.4)	1479 (92.4)	0.011
Past smoker	530 (47.7)	606 (54.6)	560 (50.4)		71 (4.4)	71 (4.4)	52 (3.3)	
Current smoker	402 (36.2)	312 (28.1)	340 (30.6)		87 (5.4)	59 (3.7)	55 (3.4)	
Alcohol consumption								
Non-drinker	282 (25.4)	409 (36.8)	416 (37.4)	<0.001	1144 (71.5)	1212 (75.7)	1195 (74.6)	0.078
Current drinker	814 (73.3)	693 (62.4)	691 (62.2)		436 (27.2)	374 (23.4)	389 (24.3)	
Physical activity								
Low	486 (43.7)	452 (40.7)	405 (36.5)	0.012	785 (49.0)	746 (46.6)	737 (46.0)	0.430
Moderate	328 (29.5)	347 (31.2)	372 (33.5)		492 (30.7)	543 (33.9)	528 (33.0)	
High	285 (25.7)	304 (27.4)	329 (29.6)		310 (19.4)	295 (18.4)	317 (19.8)	
Whole grain, bean products, and kimchi								
Age (year)	64.1 ± 9.0	64.5 ± 8.7	63.3 ± 8.0	0.005	63.9 ± 9.7	63.0 ± 9.4	62.2 ± 8.8	<0.001
BMI (kg/m^2^)	23.4 ± 3.0	23.8 ± 2.8	23.9 ± 2.9	<0.001	24.1 ± 3.3	24.3 ± 3.2	24.3 ± 3.2	0.088
Obesity prevalence, *n* (%)	326 (29.3)	353 (31.8)	377 (33.9)	0.067	578 (36.1)	622 (38.9)	599 (37.4)	0.275
Lean body mass (kg)	50.4 ± 0.3	50.3 ± 0.3	50.6 ± 0.3	0.194	36.9 ± 0.2	36.9 ± 0.2	37.1 ± 0.2	0.390
Appendicular skeletal muscle mass (kg)	20.6 ± 0.2	20.5 ± 0.2	20.6 ± 0.2	0.451	14.0 ± 1.0	14.0 ± 1.0	14.0 ± 1.0	0.805
Body fat mass (kg)	14.9 ± 0.2	15.0 ± 0.2	15.1 ± 0.2	0.305	19.4 ± 0.2	19.6 ± 0.2	19.7 ± 0.2	0.015
% Body fat (%)	22.7 ± 0.3	22.5 ± 0.3	22.0 ± 0.3	<0.001	34.3 ± 0.2	34.1 ± 0.2	33.8 ± 0.2	0.009
Serum vitamin D (ng/mL)	21.5 ± 7.1	21.0 ± 7.4	21.4 ± 7.3	0.203	18.8 ± 7.0	18.6 ± 6.7	18.6 ± 6.9	0.571
Household income, *n* (%)								
Low	375 (33.8)	335 (30.2)	288 (25.9)	0.003	638 (39.9)	551 (34.4)	504 (31.5)	<0.001
Middle-low	284 (25.6)	288 (25.9)	301 (27.1)		424 (26.5)	404 (25.2)	378 (23.6)	
Middle-high	233 (21.0)	232 (20.9)	241 (21.7)		272 (17.0)	317 (19.8)	337 (21.1)	
High	206 (18.5)	244 (22.0)	274 (24.7)		244 (15.2)	300 (18.7)	361 (22.6)	
Current smoking status								
Non-smoker	150 (13.5)	189 (17.0)	223 (20.1)	<0.001	1397 (87.3)	1477 (92.3)	1495 (93.4)	<0.001
Past smoker	513 (46.2)	587 (52.8)	596 (53.7)		81 (5.1)	58 (3.6)	55 (3.4)	
Current smoker	438 (39.4)	328 (29.5)	288 (25.9)		109 (6.8)	53 (3.3)	39 (2.4)	
Alcohol consumption								
Non-drinker	400 (36.0)	353 (31.8)	354 (31.9)	0.083	1151 (71.9)	1179 (73.6)	1221 (76.3)	0.079
Current drinker	699 (62.9)	748 (67.3)	751 (67.6)		432 (27.0)	405 (25.3)	362 (22.6)	
Physical activity								
Low	490 (44.1)	464 (41.8)	389 (35.0)	<0.001	810 (50.6)	739 (46.2)	719 (44.9)	0.007
Moderate	341 (30.7)	333 (30.0)	373 (33.6)		460 (28.7)	540 (33.7)	563 (35.2)	
High	267 (24.0)	306 (27.5)	345 (31.1)		314 (19.6)	304 (19.0)	304 (19.0)	

T: tertile, BMI: Body mass index. ^1^ *p* values were calculated using a generalized linear model for continuous variables and χ2 test for categorical variables; ^2^ Body composition variables, including lean body mass, appendicular skeletal muscle mass, body fat mass, % body fat, were presented as least squares means (LSmeans) ± standard errors adjusted for age (continuous), BMI (continuous), energy intake (continuous), smoking status (never, past, current), alcohol consumption (non-current, current), physical activity (low, moderate, and high), household income (low, middle-low, middle-high, and high), and serum vitamin D (continuous); ^3^ Numbers of missing values were 105, 60, 81 and 75 for household income, current smoking status, alcohol consumption, and physical activity, respectively.

**Table 2:** Nutrient intakes of study participants according to the tertiles of each dietary pattern score by sex.

	Tertile of Each Pattern Score							
	Men				Women			
	T1	T2	T3	*p* Value	T1	T2	T3	*p* Value
Condiment, vegetables, and meats								
Energy intake (kcal)	1522.7 ± 406.5	1981.9 ± 464.8	2703.1 ± 685.4	<0.001	1201.8 ± 395.5	1516.9 ± 413.2	1980.7 ± 558.7	<0.001
% energy from carbohydrate	77.1 ± 8.7	69.8 ± 10.5	59.2 ± 13.3	<0.001	80.4 ± 7.6	76.1 ± 8.5	68.6 ± 10.8	<0.001
% energy from protein	12.0 ± 3.0	13.9 ± 3.5	15.2 ± 4.0	<0.001	11.5 ± 2.9	13.1 ± 3.2	15.0 ± 4.0	<0.001
% energy from fat	9.8 ± 5.5	13.0 ± 6.0	18.1 ± 7.9	<0.001	9.0 ± 5.7	11.9 ± 6.0	16.9 ± 7.4	<0.001
Fiber (g)	6.2 ± 3.9	8.6 ± 5.2	10.4 ± 6.5	<0.001	5.1 ± 4.5	7.1 ± 5.3	9.2 ± 6.0	<0.001
Calcium (mg)	363.6 ± 235.8	538.2 ± 335.6	702.3 ± 407.0	<0.001	285.6 ± 212.6	413.2 ± 251.5	573.8 ± 469.1	<0.001
Phosphorus (mg)	893.2 ± 296.4	1213.4 ± 358.3	1625.8 ± 498.2	<0.001	695.7 ± 271.5	919.4 ± 293.7	1251.0 ± 430.4	<0.001
Iron (mg)	10.7 ± 7.5	16.4 ± 12.0	21.7 ± 15.6	<0.001	8.9 ± 13.5	12.8 ± 8.7	18.2 ± 16.3	<0.001
Sodium (mg)	3503.2 ± 2039.7	5306.3 ± 2786.6	7188.4 ± 3491.6	<0.001	2391.9 ± 1493.8	3786.6 ± 2111.2	5355.5 ± 2968.2	<0.001
Potassium (mg)	2254.2 ± 1055.4	3180.4 ± 1267.5	4156.0 ± 1535.6	<0.001	1880.7 ± 1198.0	2533.5 ± 1182.8	3477.9 ± 1496.2	<0.001
Vitamin A (μgRE)	505.8 ± 643.3	787.4 ± 709.6	1100.0 ± 922.8	<0.001	427.4 ± 651.2	637.4 ± 680.9	990.3 ± 1002.1	<0.001
Carotenoid (μg)	2735.4 ± 3377.0	4289.4 ± 4134.8	5681.9 ± 4606.1	<0.001	2367.6 ± 3874.4	3524.4 ± 4029.1	5204.0 ± 5298.6	< 0.001
Thiamin (mg)	0.9 ± 0.4	1.2 ± 0.5	1.8 ± 0.8	<0.001	0.7 ± 0.3	0.9 ± 0.4	1.3 ± 0.6	< 0.001
Riboflavin (mg)	0.7 ± 0.4	1.1 ± 0.5	1.6 ± 0.7	<0.001	0.6 ± 0.4	0.8 ± 0.4	1.2 ± 0.6	< 0.001
Niacin (mg)	10.8 ± 3.8	16.1 ± 5.5	24.2 ± 9.2	<0.001	8.2 ± 3.6	11.5 ± 4.0	17.5 ± 7.1	< 0.001
Vitamin C (mg)	75.3 ± 70.3	106.3 ± 79.3	133.4 ± 97.4	<0.001	68.0 ± 80.2	92.4 ± 73.3	127.9 ± 98.8	< 0.001
Wheat flour, breads, fruits, milk, and dairy products								
Energy intake(kcal)	2270.8 ± 740.9	1823.0 ± 620.0	2114.0 ± 723.2	<0.001	1564.8 ± 502.9	1400.4 ± 501.1	1734.2 ± 621.9	<0.001
% energy from carbohydrate	64.8 ± 16.2	71.7 ± 11.3	69.6 ± 10.4	<0.001	76.8 ± 10.7	75.4 ± 9.4	72.9 ± 10.2	<0.001
% energy from protein	13.1 ± 4.0	13.6 ± 3.5	14.4 ± 3.7	<0.001	12.5 ± 3.6	13.3 ± 3.6	13.9 ± 3.8	<0.001
% energy from fat	11.9 ± 7.6	12.5 ± 6.2	16.5 ± 7.2	<0.001	10.0 ± 7.0	12.2 ± 6.4	15.6 ± 7.1	<0.001
Fiber (g)	8.7 ± 5.1	7.5 ± 4.6	9.0 ± 6.7	<0.001	6.6 ± 4.7	6.2 ± 4.3	8.7 ± 7.0	<0.001
Calcium (mg)	523.9 ± 383.0	467.0 ± 330.9	613.2 ± 352.5	<0.001	367.1 ± 425.7	358.5 ± 254.9	547.0 ± 318.5	<0.001
Phosphorus (mg)	1266.8 ± 485.4	1110.9 ± 443.7	1354.7 ± 521.1	<0.001	898.1 ± 361.0	848.3 ± 354.5	1119.7 ± 451.3	<0.001
Iron (mg)	16.8 ± 14.0	14.3 ± 9.8	17.7 ± 14.4	<0.001	12.1 ± 11.6	11.8 ± 10.5	16.1 ± 17.7	<0.001
Sodium (mg)	6172.2 ± 3621.6	4886.1 ± 2936.0	4939.6 ± 2849.4	<0.001	4214.6 ± 2811.5	3497.5 ± 2347.5	3821.9 ± 2494.9	<0.001
Potassium (mg)	3145.8 ± 1403.0	2840.7 ± 1328.6	3604.1 ± 1691.1	<0.001	2292.1 ± 1170.3	2304.8 ± 1158.5	3295.1 ± 1728.9	<0.001
Vitamin A (μgRE)	764.8 ± 772.8	705.2 ± 750.5	923.1 ± 871.7	<0.001	575.3 ± 674.2	605.6 ± 688.5	874.2 ± 1034.9	<0.001
Carotenoid (μg)	4166.8 ± 3937.1	3783.4 ± 3877.9	4756.4 ± 4800.6	<0.001	3197.5 ± 3715.8	3265.1 ± 3623.5	4633.4 ± 5927.9	<0.001
Thiamin (mg)	1.4 ± 0.8	1.1 ± 0.6	1.4 ± 0.6	<0.001	0.9 ± 0.5	0.9 ± 0.5	1.2 ± 0.6	<0.001
Riboflavin (mg)	1.1 ± 0.6	1.0 ± 0.6	1.4 ± 0.6	<0.001	0.7 ± 0.4	0.8 ± 0.4	1.2 ± 0.6	<0.001
Niacin (mg)	18.2 ± 9.2	15.1 ± 7.5	17.8 ± 8.6	<0.001	11.8 ± 5.9	11.3 ± 5.8	14.2 ± 7.1	<0.001
Vitamin C (mg)	98.3 ± 71.6	94.9 ± 87.2	121.8 ± 96.2	<0.001	74.8 ± 59.0	84.6 ± 72.9	128.8 ± 113.7	<0.001
White rice, fish, and seaweeds								
Energy intake(kcal)	2103.1 ± 812.4	1819.5 ± 598.4	2285.1 ± 656.9	<0.001	1379.2 ± 573.4	1429.1 ± 438.7	1891.1 ± 515.6	<0.001
% energy from carbohydrate	60.4 ±15.0	71.9 ± 10.2	73.8 ± 9.5	<0.001	71.1 ± 11.8	76.6 ± 8.8	77.4 ± 8.8	<0.001
% energy from protein	13.8 ± 3.9	13.5 ± 3.5	13.9 ± 3.8	0.054	13.8 ± 3.8	12.9 ± 3.5	13.0 ± 3.8	<0.001
% energy from fat	16.3 ± 8.5	12.6 ± 6.6	12.1 ± 6.0	<0.001	15.2 ± 8.2	11.6 ± 6.5	11.0 ± 6.1	<0.001
Fiber (g)	7.6 ± 4.6	7.7 ± 5.2	9.9 ± 6.4	<0.001	6.4 ± 5.6	6.6 ± 5.5	8.5 ± 5.3	<0.001
Calcium (mg)	470.9 ± 300.2	484.8 ± 336.8	648.5 ± 410.2	<0.001	363.9 ± 254.9	389.0 ± 268.6	519.7 ± 468.1	<0.001
Phosphorus (mg)	1149.4 ± 481.1	1115.8 ± 433.2	1467.2 ± 490.0	<0.001	832.9 ± 401.7	876.6 ± 341.2	1156.6 ± 402.4	<0.001
Iron (mg)	14.4 ± 9.9	14.8 ± 12.6	19.6 ± 15.2	<0.001	11.4 ± 16.1	11.7 ± 8.5	16.8 ± 14.7	<0.001
Sodium (mg)	5291.9 ± 3229.7	4647.0 ± 2897.1	6059.0 ± 3330.3	<0.001	3542.0 ± 2522.2	3289.5 ± 2277.3	4702.6 ± 2685.9	<0.001
Potassium (mg)	2938.6 ± 1398.3	2866.9 ± 1418.1	3785.1 ± 1549.4	<0.001	2349.4 ± 1449.5	2382.4 ± 1261.9	3160.3 ± 1499.5	<0.001
Vitamin A (μgRE)	685.3 ± 651.5	681.6 ± 739.8	1026.2 ± 946.3	<0.001	550.2 ± 669.7	580.4 ± 622.1	924.5 ± 1063.9	<0.001
Carotenoid (μg)	3528.2 ± 3397.5	3595.0 ± 3494.6	5583.5 ± 5251.7	<0.001	2834.0 ± 3311.8	3144.2 ± 3550.0	5117.8 ± 6062.1	<0.001
Thiamin (mg)	1.4 ± 0.8	1.1 ± 0.6	1.4 ± 0.6	<0.001	0.9 ± 0.6	0.9 ± 0.5	1.1 ± 0.5	<0.001
Riboflavin (mg)	1.1 ± 0.6	1.0 ± 0.6	1.3 ± 0.6	<0.001	0.8 ± 0.5	0.8 ± 0.5	1.1 ± 0.6	<0.001
Niacin (mg)	16.7 ± 9.2	14.7 ± 7.0	19.7 ± 8.7	<0.001	11.3 ± 6.6	11.2 ± 5.5	14.8 ± 6.5	<0.001
Vitamin C (mg)	90.3 ± 71.7	97.0 ± 91.4	127.7 ± 90.2	<0.001	85.2 ± 81.5	86.9 ± 73.1	116.1 ± 103.9	<0.001
Whole grain, bean products, and kimchi								
Energy intake(kcal)	1987.6 ± 737.7	1985.9 ± 675.8	2234.2 ± 719.8	<0.001	1466.6 ± 556.8	1490.3 ± 521.0	1742.4 ± 564.3	<0.001
% energy from carbohydrate	68.9 ± 13.0	68.7 ± 13.6	68.5 ± 13.0	0.738	74.8 ± 10.8	75.4 ± 10.2	74.8 ± 9.8	0.142
% energy from protein	12.9 ± 3.9	13.6 ± 3.7	14.6 ± 3.5	<0.001	12.4 ± 3.9	13.0 ± 3.6	14.2 ± 3.4	<0.001
% energy from fat	13.2 ± 7.2	13.3 ± 7.4	14.5 ± 7.4	<0.001	12.0 ± 7.5	12.4 ± 7.2	13.3 ± 6.9	<0.001
Fiber (g)	6.1 ± 3.7	7.9 ± 5.4	11.1 ± 6.0	<0.001	5.0 ± 4.2	6.8 ± 5.0	9.7 ± 6.2	<0.001
Calcium (mg)	431.7 ± 326.9	507.3 ± 328.3	665.2 ± 385.0	<0.001	341.5 ± 413.7	402.8 ± 273.2	528.3 ± 326.1	<0.001
Phosphorus (mg)	1100.5 ± 475.0	1181.0 ± 458.1	1451.0 ± 481.2	<0.001	816.3 ± 367.6	894.7 ± 358.5	1155.2 ± 418.1	<0.001
Iron (mg)	13.2 ± 11.6	15.3 ± 10.2	20.4 ± 15.5	<0.001	10.3 ± 11.5	12.4 ± 9.2	17.2 ± 18.0	<0.001
Sodium (mg)	4387.4 ± 2581.3	5100.7 ± 2787.9	6509.8 ± 3760.9	<0.001	3127.1 ± 2171.8	3697.9 ± 2246.4	4709.0 ± 2975.0	<0.001
Potassium (mg)	2662.9 ± 1266.7	3028.8 ± 1376.9	3899.0 ± 1606.7	<0.001	2033.2 ± 1102.5	2489.8 ± 1257.9	3369.2 ± 1625.7	<0.001
Vitamin A (μgRE)	634.2 ± 674.9	757.6 ± 707.4	1001.3 ± 959.1	<0.001	497.5 ± 622.0	665.2 ± 764.3	892.4 ± 1002.1	<0.001
Carotenoid (μg)	3206.7 ± 3143.1	4050.9 ± 3674.1	5449.1 ± 5302.8	<0.001	2573.1 ± 3097.4	3576.4 ± 4082.6	4946.5 ± 5855.6	<0.001
Thiamin (mg)	1.1 ± 0.6	1.2 ± 0.6	1.5 ± 0.7	<0.001	0.8 ± 0.5	0.9 ± 0.5	1.2 ± 0.6	<0.001
Riboflavin (mg)	1.0 ± 0.6	1.1 ± 0.6	1.3 ± 0.7	<0.001	0.7 ± 0.5	0.9 ± 0.5	1.1 ± 0.6	<0.001
Niacin (mg)	16.0 ± 8.7	16.2 ± 7.9	18.9 ± 8.8	<0.001	11.1 ± 6.3	11.8 ± 6.2	14.4 ± 6.3	<0.001
Vitamin C (mg)	77.5 ± 63.3	103.9 ± 91.3	133.6 ± 92.1	<0.001	67.3 ± 62.1	92.5 ± 78.7	128.4 ± 107.0	<0.001

T: tertile.

**Table 3:** Odds ratios and 95% confidence intervals for risk of low muscle mass according to the tertiles of each dietary pattern score by sex.

	Tertile of Each Pattern Score							
	Men				Women			
	T1	T2	T3	*p* for Trend ^1^	T1	T2	T3	*p* for Trend ^1^
Condiment, vegetables, and meats								
Class I low muscle mass ^2^								
Cases/*n*	402/1111	361/1111	367/1111		465/1601	470/1601	454/1601	
OR (95% CI) ^3^	1.00	0.97 (0.78–1.20)	1.23 (0.93–1.61)	0.104	1.00	1.02 (0.85–1.21)	1.11 (0.90–1.37)	0.302
Class II low muscle mass ^2^								
Cases/*n*	169/1111	148/1111	128/1111		188/1601	157/1601	159/1601	
OR (95% CI) ^3^	1.00	1.25 (0.94–1.65)	1.51 (1.05–2.18)	0.030	1.00	0.87 (0.68–1.13)	1.20 (0.89–1.61)	0.156
Wheat flour, breads, fruits, milk, and dairy products								
Class I low muscle mass								
Cases/*n*	360/1111	390/1111	380/1111		435/1601	489/1601	465/1601	
OR (95% CI)	1.00	0.97 (0.79–1.20)	0.98 (0.79–1.20)	0.825	1.00	1.10 (0.93–1.31)	1.12 (0.94–1.34)	0.239
Class II low muscle mass								
Cases/*n*	129/1111	153/1111	163/1111		163/1601	184/1601	157/1601	
OR (95% CI)	1.00	0.97 (0.73–1.29)	1.09 (0.82–1.44)	0.483	1.00	1.06 (0.83–1.35)	1.10 (0.85–1.42)	0.487
White rice, fish, and seaweeds								
Class I low muscle mass								
Cases/*n*	405/1111	379/1111	346/1111		502/1601	463/1601	424/1601	
OR (95% CI)	1.00	0.81 (0.66–0.99)	0.72 (0.59–0.88)	0.001	1.00	0.84 (0.71–1.00)	0.76 (0.63–0.91)	0.003
Class II low muscle mass								
Cases/*n*	157/1111	164/1111	124/1111		181/1601	168/1601	155/1601	
OR (95% CI)	1.00	0.89 (0.68–1.16)	0.77 (0.58–1.02)	0.067	1.00	0.92 (0.72–1.17)	0.93 (0.71–1.22)	0.568
Whole grain, bean products, and kimchi								
Class I low muscle mass								
Cases/*n*	373/1111	393/1111	364/1111		445/1601	451/1601	493/1601	
OR (95% CI)	1.00	1.06 (0.87–1.29)	0.97 (0.79–1.19)	0.677	1.00	0.97 (0.82–1.15)	1.17 (0.98–1.39)	0.058
Class II low muscle mass								
Cases/*n*	131/1111	168/1111	146/1111		168/1601	175/1601	161/1601	
OR (95% CI)	1.00	1.21 (0.92–1.58)	1.14 (0.86–1.52)	0.420	1.00	1.01 (0.79–1.28)	0.99 (0.77–1.27)	0.903

T: tertile; OR: odds ratio; CI: confidence interval; **^1^** Linear trends across tertiles of each pattern were tested using the median value for each tertile as a continuous variable; ^2^ By definition, class I low muscle mass was indicated by definition in participants whose weight-adjusted ASMM was between the gender-specific mean for young reference group-1 SD and the mean for young adults-2 SD. Class II low muscle mass was indicated by definition in participants whose weight-adjusted ASMM was below the mean for young adults-2 SD. ^3^ Adjusted for age (continuous), BMI (continuous), energy intake (continuous), smoking status (never, past, current), alcohol consumption (non-current, current), physical activity (low, moderate, and high), household income (low, middle-low, middle-high, and high), and serum vitamin D (continuous).
